# Environmental Impacts of Microplastics and Nanoplastics: A Current Overview

**DOI:** 10.3389/fmicb.2021.768297

**Published:** 2021-12-15

**Authors:** Ayodeji Amobonye, Prashant Bhagwat, Sindhu Raveendran, Suren Singh, Santhosh Pillai

**Affiliations:** ^1^Department of Biotechnology and Food Science, Faculty of Applied Sciences, Durban University of Technology, Durban, South Africa; ^2^Microbial Processes and Technology Division, CSIR-National Institute for Interdisciplinary Science and Technology (CSIR-NIIST), Trivandrum, India

**Keywords:** biodegradation, ecosystems, environment, microplastics, nanoplastics, toxicity

## Abstract

The increasing distribution of miniaturized plastic particles, *viz.* microplastics (100 nm–5 mm) and nanoplastics (less than 100 nm), across the various ecosystems is currently a subject of major environmental concern. Exacerbating these concerns is the fact that microplastics and nanoplastics (MNPs) display different properties from their corresponding bulk materials; thus, not much is understood about their full biological and ecological implications. Currently, there is evidence to prove that these miniaturized plastic particles release toxic plastic additives and can adsorb various chemicals, thereby serving as sinks for various poisonous compounds, enhancing their bioavailability, toxicity, and transportation. Furthermore, there is a potential danger for the trophic transfer of MNPs to humans and other higher animals, after being ingested by lower organisms. Thus, this paper critically analyzes our current knowledge with regard to the environmental impacts of MNPs. In this regard, the properties, sources, and damaging effects of MNPs on different habitats, particularly on the biotic components, were elucidated. Similarly, the consequent detrimental effects of these particles on humans as well as the current and future efforts at mitigating these detrimental effects were discussed. Finally, the self-cleaning efforts of the planet *via* a range of saprophytic organisms on these synthetic particles were also highlighted.

## Introduction

Globally, there has been an exponential increase in the quantity of plastics produced for their various industrial applications; consequently, there is also an equally astronomical increase in the wastes generated from these polymers. It was recently estimated that the manufacture of plastic had grown astronomically from a few tons in the early 1950s to close to 400 million tons in 2018 (Syberg et al., 2021). Plastic wastes have since been recognized as pervasive, relatively non-biodegradable, and widely distributed across different ecosystems on our planet (Amobonye et al., 2020). Thus, it has become a growing source of concern and discourse among biologists, conservationists, environmentalists, as well as the general public. Furthermore, it has been noted that over time, plastic wastes eventually lose their mechanical integrity through various environmental factors including abrasion, photooxidation, as well as various biotic degradation pathways (Wright et al., 2020). In due course, these waste plastics are miniaturized to minute particles, *viz.* microplastics and nanoplastics (MNPs), which also pose an immeasurable danger to the environment (Hartmann et al., 2019). These “smaller” plastic particles came to the scientific spotlights in the 1970s after being identified as a major component of the ocean floor debris and even gained more attention when the term “microplastics” was introduced. The term that was coined by Thompson et al. (2004) to initially describe the accumulation of microscopic plastic particles in marine sediments and in the water column of European waters has since been broadened to include both MNPs, which are small pieces of plastic that arise from the fragmentation of larger pieces.

Currently, the literature has been observed to be inconsistent about the categorization of the particle sizes of MNPs as their particle size ranges have been defined differently by various authors and organizations. However, it was observed that many studies had set the upper limits for microplastics at 5 mm (Frère et al., 2018; Cai et al., 2020a; Stock et al., 2021), while the upper limit for nanoplastics was set at 100 nm (Lehner et al., 2019; Gonçalves and Bebianno, 2021a). Furthermore, the International Organization for Standardization has also defined nanoparticles as objects with their external dimensions existing in the nanoscale (size range from approximately 1 to 100 nm; Boverhof et al., 2015). Hence in this study, microplastics are specifically defined as plastic particles within the 100 nm–5 mm size range, while nanoplastics are particles of plastic origin that are less than 100 nm in size. Both MNPs have been found to dominate plastic particle counts in various environments. In this regard, they have been reported to be present all around the planet, from polar regions frozen in ice to the open water around the equator, as well as from coastline down to the deep sea.

Microplastics and nanoplastics have been categorized according to their sources into primary and secondary microplastics/nanoplastics. Primary MNPs are the by-products of particulate emissions released from different industrial production and enter the environment in their original small sizes, which are associated with their specific applications and consumer products (Gonçalves and Bebianno, 2021b). These products have been identified to mainly include cosmetic and cleaning products such as toothpaste, raw materials used for plastic goods manufacture as well as textile fibers released during washing or drying (da Costa, 2018). Secondary MNPs, on the other hand, are formed as a result of plastic debris degradation due to their exposure to physical, animal, and microbial (Laskar and Kumar, 2019).

Although most MNPs are derived from polymeric bulk materials that have been demonstrated to be relatively biochemically inert, alterations in the physicochemical properties of MNPs have enhanced their bioavailability and toxicity (Ma et al., 2020). Hence, MNPs’ contamination in both aquatic and terrestrial environments has been the focus of numerous scientific investigations due to their ubiquitous distribution and potential risks to living organisms.

Microplastics and nanoplastics have been recognized majorly as marine contaminants with estimates of hundreds of thousand metric tons floating on the surfaces of the major marine ecosystems (Wright et al., 2020). However, recent studies have also shown MNPs permeating freshwater bodies (Li et al., 2020a; Dong et al., 2021) and various terrestrial environments (Mohajerani and Karabatak, 2020; Tan et al., 2021). Furthermore, due to the heterogeneity of MNPs and various inorganic plastic particles as well as organic matter, they have the propensity to form both homo- or hetero-aggregates as highlighted in many studies (Jakubowicz et al., 2020; Sharma et al., 2021). The formation of these undesirable aggregates leads to the bioaccumulation and bioamplification phenomena causing detrimental effects to the biotic components in different ecosystems (da Costa, 2018).

The high ubiquity of MNPs has grave environmental consequences that transverse national boundaries. This is further worsened by the fact that they largely exhibit multi-scalar, temporal reaction and absorption mechanisms, which are still not yet well understood. However, it has now been shown that MNPs can be ingested by numerous organisms because of their minute sizes, raising more health concerns, especially with regard to their potential to disrupt cellular membranes (Ding et al., 2021) and cause oxidative stress (Hu and Palić, 2020). Marine organisms such as bivalves, copepods, echinoderms as well as polychaetes have been noted to have high probabilities of taking up MNPs from the environment, at least once in their different life stages (Yu et al., 2020; Zhang et al., 2020b; Suckling, 2021). In addition, MNPs also act as pollutant transport media for other toxic elements, such as DDT and hexachlorobenzene. Consequently, these toxic elements find their way into the systems of living organisms through MNPs’ consumption and may result in progression along different food chains, ultimately affecting human health (Jin et al., 2021). The atmosphere (both indoor and outdoor) has also been identified as a new vehicle for MNPs’ transportation across environments, raising public health concerns due to the potential exposure (Evangeliou et al., 2020; Chen et al., 2020a).

However, there are still many grey areas concerning the chronic effects of MNPs’ exposure, as well as the eventual implications of their contamination in different ecosystems. In this regard, this paper seeks to shed more light on the environmental fate and impacts of MNPs. The paper critically reviews recent investigations on the sources of MNPs, their occurrence and effects on the abiotic and biotic components of various ecosystems, and recent efforts at mitigating their detrimental effects on life forms. It is expected that information from this article will serve as an important reference for all relevant stakeholders, including scientists and policymakers, in charting a new course for the effective management of MNPs and their attendant effects.

## Methodology

The review process was initiated by internet searches using the ISI Web of Science,^1^ PubMed,^2^ ScienceDirect,^3^ and Scopus^4^ databases. The searches were conducted using the following keywords and strings: (Microplastics OR Nanoplastics) AND (environment OR ecosystems OR habitats OR NATURE) AND (biodegradation OR degradation OR breakdown) AND (effects OR consequence OR actions) AND (mechanism OR steps OR processes) AND (reduction OR mitigation OR remediation). This procedure allowed the filtering of published works on the effects, sources, and occurrence of MNPs as well as efforts at mitigating their effects. Publications on macroplastics were excluded, and focus was placed on MNPs’ articles within the last 10 years except for historical purposes or in cases where there is a lack of recent literature on the discourse. Two independent searches were done, and the conformity of the chosen papers was validated based on the inclusion criteria described. In addition, articles from predatory/unreputable journals, unpublished literature and publications in languages besides English were not considered for this review. Finally, data from the search results were analyzed, categorized, and presented under the appropriate sections to cover the scope of this article.

## Classification and Sources of Mnps

**Figure 1:**
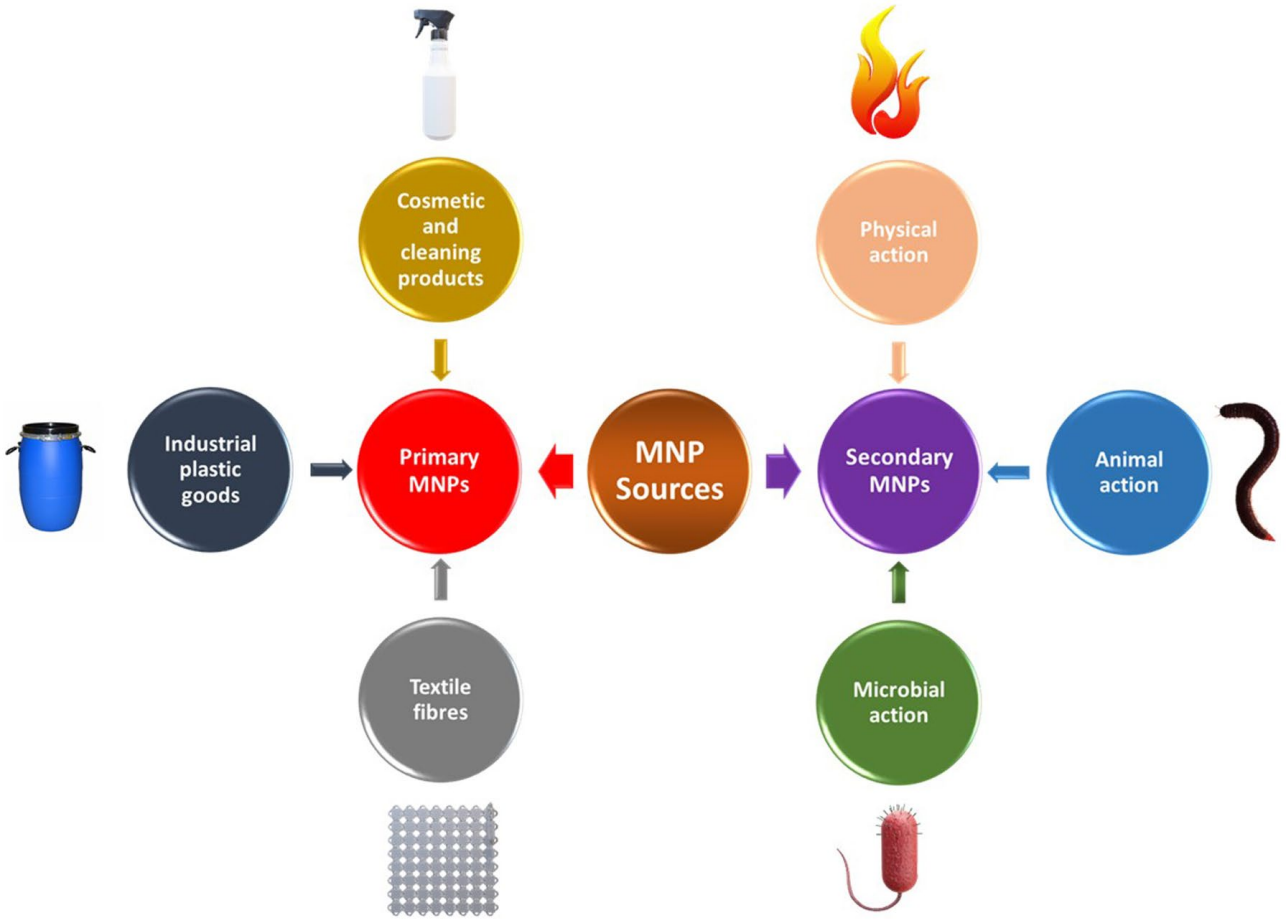
Primary and secondary sources of MNPs.

As earlier stated, MNPs are either directly manufactured or derived from the fragmentation of larger plastics over time; hence, it is on this basis that they are generally classified into primary and secondary MNPs as highlighted in Figure 1. Primary MNPs are basically sourced from plastic pellets and microbead-containing personal care products. Other important sources of primary MNPs include artificial turfs, paints, washed textile and wastewater, sewage sludge, plastic running tracks in schools, rubber roads as well vehicle tire wear (Smyth et al., 2021). Microbeads are special microplastics mostly made from polyethylene, polypropylene, and polystyrene, which are intentionally added to cosmetics and other personal care products (Guerranti et al., 2019). They specifically serve as exfoliating and scrubbing agents in personal care products as well as in biomedical and health-science research (Dalili et al., 2019). MNP microbeads are also used in personal care products and cosmetics as film-forming agents, functionalized polymers, hydrophilic agents, and silicones (Lochhead, 2017). Their properties such as sphericity and particle size uniformity create a ball-bearing effect, which results in silky texture and spreadability, which are desirable qualities in cosmetics (Alves et al., 2020). These MNPs are not only spherical but could also be elliptical, irregularly frayed, and thread-like and can also be used instead of natural materials like pumice stone and activated carbon (Napper et al., 2015). Colored microbeads also add visual appeal to personal care products. They have been identified as a problematic source of microplastics as they pass unhindered through sewage treatment plants after being washed down the drain, ending up in canals, rivers, streams, and other water bodies (Ding et al., 2020b). It was estimated that 11% (~ 2,300 t/a) of the plastic waste discharged into the North Sea are microbeads from MNPs (Brzuska et al., 2015). Pre-production resin pellets (granulate) mainly used in industrial plastic manufacture have also been noted to be a major source of MNPs debris. These plastic pellets are also products of plastic recycling, particularly during the cleaning, crushing, melting, sorting, and final molding processes (Duncan et al., 2018).

Secondary MNPs are produced from the fragmentation of macroplastics materials *via* different environmental degradation processes such as biodegradation, chemical (corrosion, photooxidation, temperature), as well as mechanical (abrasion erosion, wave action) activities (Karbalaei et al., 2018). In this regard, the major sources of secondary MNPs have been noted to include municipal wastes such as farming films, plastic bags and bottles, fishing gears, shipping, vehicle tire wear, and other large size plastic wastes. It was recently estimated that secondary sources of MNPs account for most of the MNPs in both the aquatic and terrestrial environments (An et al., 2020). Vehicle tire wear and road marking abrasions are particularly regarded as one of the most prevalent sources of environmental MNPs due to the increasing volume of vehicles worldwide (An et al., 2020; Kitahara and Nakata, 2020). Similarly, synthetic textile fibers have been observed to shed MNPs during laundering in large quantities, which eventually find their way into water bodies and/or wastewater treatment plants (Galvão et al., 2020). A recent study by De Falco et al. (2019) revealed that approximately 124 to 308 mg of microplastics, which corresponds to 640,000–1,500,000 MNPs particles, is released per kg of washed fabric depending on the type of textile material. The construction industry also releases a considerable amount of MNPs from plastic polymers used in cladding, insulating materials and pipes; however, it is believed that MNPs are mostly produced on construction sites as a result of carelessness or improper storage (Battulga et al., 2019). MNPs are also used in special applications such as blasting agents in sandblasting for paint stripping, cleaning, roughening or refining surface (Battulga et al., 2019). A summary of the major sources of MNPs and their entry routes into the various ecosystems is presented in Table 1.

**Table 1:** Sources of microplastics and nanoplastics into the environment.

MNPs’ source	Properties	Entry point into the environment	Application	References
Plastic pellets	Granular plastics, commonly with a diameter of 2–5 mm and a regular shape	Drifting/surface runoff/loss	Raw materials and building blocks for nearly every plastic product.	Karkanorachaki et al. (2018); Mendoza et al. (2018)
Microbead personal care products	Microbeads varying in color and ~ 100–1,000 μm	Wastewater/sewage sludge	Exfoliating, film-forming, hydrophilic, scrubbing agents and functionalized polymers in personal care products as well as in biomedical applications.	Napper et al. (2015); Nel et al. (2019)
Paint	Between 0.3 and 5 mm from synthetic polymers mainly alkyds, epoxy resins, poly(acrylate/styrene) and polyurethane	Surface runoff	Architectural coatings, marine coatings, automotive coatings, and road-marking paint.	An et al. (2020); Gaylarde et al. (2021)
Textile fabric	100–1,000 μm MNPs mainly from acrylic, polyethylene terephthalate, and nylon fabrics	Wastewater/sewage sludge	Enhanced appeal and functionality in synthetic fabrics.	Carney Almroth et al. (2018); Cai et al. (2020b)
Sewage treatment effluents	Different kinds of MNPs from automobile tire wear, industrial production of plastic, personal care products, chemical laundry products, urban debris, etc. Ranging from 0.1 μm to 5 mm	N/A	N/A	Domogalla-Urbansky et al. (2019); Mak et al. (2020)
Sports ground (artificial turfs and running tracks)	MNPs from propylene, polyamide 6 (PA6), PE, or polyurethane, styrene-butadiene rubber, thermoplastic elastomer, and green rubber and ethylene propylene diene monomer which is made of EPDM. Usually between 0.5–2.5 mm	Drifting/surface runoff	N/A	Wang et al. (2019); van Kleunen et al. (2020)
Vehicle tire wear	Roundish, kidney-shaped or elongated particles from styrene butadiene rubber and natural rubber particles 0.01–350 μm	Surface runoff	N/A	Sommer et al. (2018); Järlskog et al. (2020)
Municipal debris	Fragments of plastic bags, plastic bottles and other packaging materials. Of varying particle sizes from 0.1 μm to 5 mm	Drifting/surface runoff	N/A	Welle and Franz (2018); Sobhani et al. (2020)
Fishing gears	Polyethylene, polyamide (nylon), and polypropylene monofilaments of between 0.1 and 5 mm	Drifting/loss	N/A	Dowarah and Devipriya (2019); Xue et al. (2020)
Farming films	Microfilms more commonly from polyethylene between 0.03 and 10 mm	Drifting/surface runoff	N/A	Liu et al. (2018); Zhang and Liu (2018)
Construction industry	Fragments of typically polyamide, polyethylene, polyvinylchloride and polyurethane polymers	Drifting/surface runoff	N/A	Dehghani et al. (2017); Xu et al. (2020)

*N/A—Not applicable.*

## Occurrence and Effects of Mnps in Aquatic Habitats

MNPs’ pollution is ubiquitous in aquatic (marine and freshwater), terrestrial, and atmospheric environmental compartments, which are interconnected *via* diverse networks of source–pathway–sink connections that can influence MNPs’ flux and retention (Bergmann et al., 2019; Zhang et al., 2020d). However, most of the studies done in the last decade have primarily concentrated on aquatic habitats for MNPs in comparison with land and the atmosphere. MNPs debris in water bodies is composed mainly of different types of plastics, including polyethylene (PE), polypropylene (PP), polystyrene (PS), and polyvinyl chloride, which interact with organic matter, inorganic elements, and microorganisms in aquatic habitats (Mammo et al., 2020). In addition, several studies have also demonstrated that MNPs could adsorb various toxic chemicals. Thus, it is now widely accepted that MNPs can act as a magnet for toxic chemicals in the environment and transport them within and between different habitats (Wu et al., 2019; Yuan et al., 2020). Ultimately, the MNPs could be consumed by diverse marine species, thereby slowly entering the marine food web and posing severe threats to all marine and terrestrial life (Al-Thawadi, 2020).

The overall abundance and physicochemical characteristics of MNPs in aquatic (marine and freshwater) habitats varied significantly across the various study areas (Table 2). For example, in a recent study, the distribution of microplastic contamination in two developed estuaries in the South-Eastern United States was investigated (Gray et al., 2018). Microplastic concentrations in the sea surface microlayer of Charleston Harbour ranged from 3 to 11 particles/l, with an average of 6.6 ± 1.3 particles/l, and in the sea surface microlayer of Winyah Bay, the microplastic concentrations ranged from 6 to 88 particles/l, with an average of 30.8 ± 12.1 particles/l (Gray et al., 2018). Lower MNPs’ abundance in marine habitats was, however, recorded in different studies, for example, in Barents, Kara, Laptev, and East-Siberian Seas (0–0.045 particles/m^2^; Yakushev et al., 2021), Lake Winnipeg, Canada (0.19 particles/m^2^; Anderson et al., 2017), Gullmar Fjord, Swedish West Coast (0.18–0.92 particles/m^2^; Karlsson et al., 2020), Skagerrak/Kattegat, Baltic Sea and Gulf of Bothnia (3.74 particles/m^2^; Schönlau et al., 2020), to name a few.

**Table 2:** Abundance and physicochemical characteristics of MNPs in aquatic habitats.

Sample/study region	MNPs abundance	MNPs shape	MNPs size	Method	Composition	References
Lake Winnipeg, Canada	0.19 particles/m^2^	Fibers 90% Films Foams	< 5 mm	Dissecting microscope Scanning electron microscope (SEM) Energy-dispersive X-ray spectroscopy (EDX)	NA	Anderson et al. (2017)
Charleston Harbor USA	3–11 particles/l	Fibers Foams Spheres	63–500 μm	Attenuated total reflectance–Fourier transform infrared spectroscopy (ATR-FTIR)	Polystyrene PA Polyester PE PP	Gray et al. (2018)
Winyah Bay USA	6–88 particles/l	Fibers Foams Spheres	63–500 μm	ATR-FTIR	Polystyrene PA Polyester PE PP	Gray et al. (2018)
Gullmar fjord Swedish west coast	0.18–0.92 particles/m^2^	Fibers Spheres	300–500 μm	FTIR	Polystyrene (PS) Polymethylmethacrylate (PMMA) Polypropylene (PP) polyamide (PA)	Karlsson et al. (2020)
Coastline Tamil Nadu, India	2–178 particles/m^2^	Fibers 24–27 Fragments 47–50% Foams 10–19	0.3–4.75 mm	Attenuated total reflectance (ATR) FTIR (ATR-FTIR)	PP PE	Karthik et al. (2018)
*Mytilus edulis* Coastal waters of China	0.9–4.6 particles/g	Fibers Spheres Flakes Fragments	<250 μm	Stereo microscope μ-FTIR	Cellophane (CP) Polyethylene terephthalate (PET) Polyester (PES)	Li et al. (2016)
Drinking water treatment plants North-western part of Germany	0–7 particles/m^2^ (raw water) 0.7 particles/m^2^ (treated water)	Fibers Fragments	50–150 μm	μ-FTIR	PA PE PVC Polyester	Mintenig et al. (2019)
Sea ice Fram Strait Central Arctic	1.1–12 × 10^6^ N/m^3^	Fibers	11–50 μm	Focal plane array (FPA) FTIR microscopy	Cellulose acetate Ethylene vinyl acetate (EVA) PA PE Polyester (PES) Polypropylene (PP) Varnish	Peeken et al. (2018)
Changjiang Estuary China	20–340 particles/kg	Fibers Pellets	46.8–4968.7 μm	μ-FTIR	Rayon Polyester Acrylic	Peng et al. (2017)
Rivers and tidal flat Shanghai, China	52–1,600 particles/kg	Fibers Fragments	<100–500 μm	μ-FTIR	PP Rayon Polyester	Peng et al. (2018)
Drinking water treatment plants Czech Republic	1,473–3,605 particles/l (raw water) 338–628 particles/l (treated water)	Fibers Fragments	1–10 μm	FTIR Raman spectroscopy SEM–EDX	PET PE PP	Pivokonsky et al. (2018)
Skagerrak/Kattegat, Baltic Sea and Gulf of Bothnia	3.74 particles/m^2^	Fibers	50–300 μm	Near-infrared (NIR) hyperspectral imaging	PE, PP, PS, and polyamide (PA)	Schönlau et al. (2020)
Drinking bottled waters Germany	193 particles/l	Fibers Fragments	1–20 μm	micro-Raman spectroscopy	PET PP	Schymanski et al. (2018)
Seawater North Atlantic subtropical gyre	13–501 particles/m^2^	Fibers	1–1,000 nm	Dynamic light scattering (DLS) FTIR Pyrolysis coupled with gas chromatography–mass spectrometry	PVC PET PS PE	Ter Halle et al. (2017)
Hanjiang River and Yangtze River Wuhan, China	1,660–8,925 n/m^3^	Fibers Granule Films Pellets	50 μm–2 mm	Stereoscopic microscope SEM FTIR	PET PP	Wang et al. (2017)
Seawater Barents, Kara, Laptev and East-Siberian Seas	0–0.045 particles/m^2^	Fragments (80.5) Fibers (19.5%)	< 5 mm	μ-FTIR	Polyethylene (PE) Polyurethane (PUR) Polyvinyl chloride (PVC) Polyester Polyamide (PA) Polystyrene (PS) Polypropylene (PP).	Yakushev et al. (2021)
Table salts China	550–681 particles/kg (sea salts) 43–364 particles/kg (lake salts) 7–204 particles/kg (well salts)	Fibers Pellets Sheets	< 200 μm	Stereo microscope μ-FTIR	Polyethylene terephthalate (PET) Polyester (PES) Polyethylene (PE) Poly(1-butene; PB) Polypropylene (PP) Cellophane (CP)	Yang et al. (2015)
Southeastern NPS units United States	100–300 particles/kg	Fibers	~20 μm	FTIR	PET	Yu et al. (2018)

Meanwhile, concerns regarding the health repercussions of MNPs on humans have been raised due to the observations in many studies that freshwater sources are even rifer with MNPs compared to seawater. For example, significantly higher concentrations of MNPs were observed in raw (1473–3,605 particles/l) and treated water (338–628 particles/l) in a drinking water treatment plant in the Czech Republic (Pivokonsky et al., 2018). Another study conducted in Germany also found MNPs (193 particles/l) in drinking bottled waters (Schymanski et al., 2018). Similarly, many water-related habitats and points of interaction have been demonstrated to have a significant share of MNPs proliferation. These include a coastline in Tamil Nadu, India (2–178 particles/m^2^), and North Atlantic subtropical gyre (13–501 particles/m^2^; Ter Halle et al., 2017; Karthik et al., 2018). An abundance of MNPs has also been reported from sediments of Changjiang Estuary, China (20–340 particles/kg; Peng et al., 2017); rivers and tidal flat, Shanghai, China (52–1,600 particles/kg; Peng et al., 2018), and South-Eastern National Park Service (NPS) units, United States (100–300 particles/kg; Yu et al., 2018).

The detrimental effect of MNPs on aquatic life, including microbes and invertebrates such as zooplankton, as well as vertebrates such as fish, seabirds, and amphibians, has been the focus of many studies (Frydkjær et al., 2017; Sana et al., 2020). MNPs have been reported to inhibit the growth of microorganisms such as certain yeast, bacteria, and algae, thus affecting their important fundamental roles in different environments (Nomura et al., 2016; Sun et al., 2018; Mammo et al., 2020). Furthermore, MNPs have been observed to obstruct the digestive systems in zooplanktons and marine benthic organisms such as mussels and oysters, causing decreased appetite, malnutrition, and deaths in many cases (Lee et al., 2013; Van Cauwenberghe et al., 2013). MNPs also pose an additional risk to human health as they are ingested by a variety of aquatic organisms, both freshwater and marine, and thus can accumulate through the food web (Sana et al., 2020). In this regard, it has been suggested that specific bioindicator organisms be chosen as sentinel species to biomonitor the impact of MNPs in various geographical niches, thereby ensuring the safety of aquatic food products. For example, the lugworm (*Arenicola marina*), a robust deposit feeder at the base of the benthic food web, is commonly used in marine sediment toxicity tests (Besseling et al., 2017), while the mussel (*Mytilus galloprovincialis*) is an internationally recognized sentinel species for monitoring marine pollution (Al-Thawadi, 2020).

## Occurrence and Effects of Mnps in Terrestrial Habitats

MNPs’ pollution is a major contributor to one of the most pervasive and long-term anthropogenic changes transpired to the earth’s terrestrial habitat. Consequently, overwhelming evidence of direct and indirect deleterious effects of MNPs’ pollution on various terrestrial habitats has emerged in recent years (Ambrosini et al., 2019; Zhang et al., 2020a; Chen et al., 2020b; Yakushev et al., 2021). It is important to note that majority of the plastic wastes that end up in water bodies were initially produced, used, and indiscriminately discarded on land (de Souza Machado et al., 2018). Therefore, terrestrial habitats are considered huge MNPs’ reservoirs, which could provide multiple exposure pathways to the biota in terrestrial systems, potentially altering the geochemistry, resulting in environmental toxicity (Allouzi et al., 2021). Thus, much focus has been given to the routine analysis of terrestrial samples, specifically soil samples, for the presence of MNPs. The overall abundance and physicochemical characteristics of MNPs from terrestrial habitats are presented in Table 3. For example, MNPs’ abundance in agricultural soil samples from South-eastern Germany was shown to range from 0 to 1.25 particles/kg of dry soil samples with a mean abundance of 0.34 particles/kg of soil (Piehl et al., 2018). However, a slightly higher content of MNPs was recorded in another study with four different soil samples from the suburbs of Shanghai. In this study, floodplain soil had the highest MNPs content (256.7 ± 62.2 particles/kg) followed by paddy soil (190 ± 31.2 particles/kg), yellow-brown soil (155 ± 95.2 particles/kg), and farmland soil (36.6 ± 41.7 particles/kg; Liu et al., 2019d). In another study, MNPs in floodplain soils from Switzerland was found to have a mean abundance of 593 particles/kg of soil samples (Scheurer and Bigalke, 2018).

**Table 3:** Abundance and physicochemical characteristics of MNPs in terrestrial habitats.

Sample/ study region	MNPs abundance	MNPs shape	MNPs size	Method	Composition	References
Vegetable farmland Wuhan, China	320–12,560 particles/kg	Fibers Microbeads	<0.2–5 mm	Micro-Raman spectroscopy	PA PP	Chen et al. (2020b)
Agricultural fields Mellipilla, Chile	1,100–3,500 particles/kg	Fibers (97%), Pellets Films	<2 mm	Stereo microscope	Acrylic Polyester Nylon LDPE PVC	Corradini et al. (2019)
Agricultural soil Shaanxi, China	1,430–3,410 particles/kg	Fibers Granules	<5 mm	FTIR	PET PP PS PVC HDPE	Ding et al. (2020a)
Soil of industrial area Sydney, Australia	300 mg·kg^−1^–67,500 mg·kg^−1^	NA	20–40 μm	FTIR	PVC PE PS	Fuller and Gautam (2016)
Vegetated wetland Washington, USA	7,387–47,047 m^−2^	Fibers Fragments	<75 μm–5 mm	FTIR	PS PE Synthetic rubber	Helcoski et al. (2020)
Agricultural soil Xinjiang, China	80.3–1075.6 particles/kg	Films	<5 mm	μ-FTIR	PE	Huang et al. (2020)
Agriculture soil Nanjing and Wuxi, China	420–1,290 particles/kg	Fibers (38.9–65.1%) Fragment	0.02–0.25 mm	Stereo microscope	PE PP	Li et al. (2019)
Sludge samples Nanjing, China	5,553–13,460 particles/kg	Fibers (75.8–88.8%)	0.02–0.25 mm	Stereo microscope	PE PET PAN	Li et al. (2019)
Vegetable farmland Shanghai, China	136.6–256.7 particles/kg	Fibers 54 Films 7 Fragments 38 Granules	0.03–4.76 mm	μ-FTIR	PE PP	Liu et al. (2019d)
Farmland Franconia, Germany	0–1.25 particles/kg	Films (43.75%), Fragments (43.75%), Fibers	1–5 mm	FTIR	PE PP PS	Piehl et al. (2018)
Floodplain soils Switzerland	593 particles/kg	NA	<500 μm–5 mm	FTIR	PE PP PS PVC	Scheurer and Bigalke (2018)
Agricultural soil Spain	930–1,100 particles/kg	Fragment (80%) Fibers Films	150–250 μm	μFTIR	PP PVC	van den Berg et al. (2020)
Sewage sludge Spain	18,000–32,070 particles/kg	Fragment (80%) Fibers Films	NA	μFTIR	PP PVC	van den Berg et al. (2020)
Farmland Heilongjiang, China	800 particles/kg	NA	0.05–5 mm	NA	LDPE	Zhang et al. (2020a)
Vegetable farmland Wuhan, China	22,000–690,000 particles/kg	Fragment (52%) Bead (14%) Fibers (13.8%)	10–500 μm	Stereo microscope	PE PP PS PA PVC	Zhou et al. (2019)

Similarly, more recent studies have observed an increase in the rate of MNPs contaminations, which could be directly ascribed to the continuous rise in plastic pollution. For example, higher MNP content of between 80 and 3,500 particles/kg has been observed in agricultural soil samples from China (Li et al., 2019; Huang et al., 2020; Ding et al., 2020a; Zhang et al., 2020a) and Chile (Corradini et al., 2019). Even higher MNPs’ content was recorded from vegetable farmland in Wuhan, China (320–12,560 particles/kg of soil; Chen et al., 2020b), and a different sampling site in the same city (22000–690,000 particles/kg of soil; Zhou et al., 2019). Besides the typical soil samples, MNPs also accumulate in wastewater treatment plants, as demonstrated by their high content in sludge. For instance, 5,553–13,460 particles of MNPs per kg of sludge were reported from Nanjing, China (Li et al., 2019). Interestingly, it was observed that a substantial portion of the studies on the terrestrial abundance of MNPs has come from China, indicating the dearth of information and the necessity for further research in other parts of the world.

The damaging effects of accumulated MNPs on the soil systems are unquantifiable; they interact with other potentially harmful elements and organic contaminants, multiplying their potential and thus severely affecting the various terrestrial biota (Chai et al., 2020). Specifically, MNPs that could persist for hundreds of years have been noted to interact with organic matter in the soil, affecting soil physiochemical parameters and polluting groundwater, consequently reducing plant growth and overall productivity (Wahl et al., 2021). In addition, MNPs also have significant negative impacts on soil fauna, especially earthworms and nematodes, affecting their growth, reproduction, lifespan, and survival through various toxicity mechanisms, including bioaccumulation, DNA damage, genotoxicity, gut microbiota dysbiosis, histopathological damage, metabolic disorders, neurotoxicity, oxidative stress, and reproductive toxicity (Wang et al., 2021a). This will impact negatively on the natural ecological activities of these organisms, such as litter decomposition, nutrient cycling and energy flow, posing various potential environmental and health hazards (Wang et al., 2020). Furthermore, due to their high surface area-to-volume ratio and hydrophobicity, MNPs might act as transporters of pathogens and organic pollutants on land, similar to previously highlighted in aquatic habitats (Atugoda et al., 2021). Also, microorganisms attached to MNPs represent a threat to the environment by acting as a conduit for MNPs to be transferred from the soil to plants and, eventually, to other living beings *via* the food chain (Chai et al., 2020).

## Occurrence and Effects of Mnps in the Atmosphere

Recent research has identified the atmosphere as a major reservoir and source of MNPs’ contamination as they have lately been identified in the atmosphere of urban, suburban, and rural areas. It has been observed that airborne MNPs can travel long distances from MNPs source regions and accumulate in a variety of terrestrial and aquatic environmental matrices, posing various threats to the biosphere (Mbachu et al., 2020). However, the fate of MNPs is determined by the connectivity of environmental compartments, and the atmosphere is the least investigated of all the environmental compartments in terms of MNPs occurrence and spatial distribution (González-Pleiter et al., 2021). Unlike microplastics in other ecosystems, microplastics in the air can be inhaled directly and continuously, posing a serious health concern. As a result, a greater understanding of the concentration, source, and risks of MNPs in the environment is critical (Chen et al., 2020a).

To date, only a few cities and areas have conducted research on detecting airborne microplastics, including Asaluyeh County (Iran), Beijing, Dongguan and Shanghai (China), California (United States), the French Pyrenees and Paris (France), Hamburg (Germany), London, Nottingham (United Kingdom), and Surabaya (Indonesia). In this regard, the overall abundance and physicochemical characteristics of the MNPs in the atmosphere are presented in Table 4. For example, the MNPs’ concentrations from two streets in Surabaya City, Indonesia, were in the range of 132.75–174.97 particles/m^3^, indicating a significant degree of MNPs pollution in the suburb (Asrin and Dipareza, 2019). Other investigations found substantially fewer MNP particles, such as suspended atmospheric fallout in Shanghai, China, which reported a mean abundance of 4.18 particles/m^3^ (Liu et al., 2019b), and suspended dust in Asaluyeh County, Iran, which reported 0.3–1.1 particles/m^3^ (Abbasi et al., 2019). In another study, relatively low concentrations of MNPs were found in suspended atmospheric aerosols at the West Pacific Ocean, ranging from 0 to 1.37 particles/m^3^ (Liu et al., 2019c).

**Table 4:** Abundance and physicochemical characteristics of MNPs in the atmosphere.

Sample/study region	MNPs abundance	MNPs shape	MNPs size	Method	Composition	References
Suspended dust Asaluyeh County, Iran	0.3–1.1 particles/m^3^	Fibers Granules	100–1,000 μm	Fluorescence microscopy SEM/EDS	N/A	Abbasi et al. (2019)
Atmospheric dry & wet deposition Pyrenees mountains, Europe	44–249 particles/m^2^d	Fibers Films Fragments	10–5,000 μm	Stereo microscope μ-Raman	PE PET PP PS PVC	Allen et al. (2019)
Supraglacial debris Italian Alps	74.4 particles/kg of sediments	N/A	N/A	μ-FTIR	PA PE Polyester PP	Ambrosini et al. (2019)
Suspended atmospheric fallout Surabaya, Indonesia	132.75–174.97 particles/m^3^	Fibers Films Pellets	<500–5,000 μm	FTIR	Cellophane PE PET	Asrin and Dipareza (2019)
European snow Europe	190–154,000 particles/l	Fibers	11–250 μm	μ-Raman FTIR imaging	Varnish Nitrile rubber PE Polyamide rubber	Bergmann et al. (2019)
Arctic snow (wet deposition) Arctic	0–14,400 particles/l	Fibers	11–475 μm	μ-Raman FTIR imaging	Varnish Nitrile rubber PE Polyamide rubber	Bergmann et al. (2019)
Atmospheric fallout (dry & wet deposition) Dongguan, China	175–313 particles/m^2^d	Fibers Films Foams	200–4,200 μm	Stereo microscope μ-FTIR	PE PP PS	Cai et al. (2017)
Urban dust Tehran metropolis, Iran	2,933–20,167 particles/kg of dry dust	Granules ~60% Fibers ~35% Sphere ~5%	100–1,000 μm	Fluorescence microscopy SEM/EDS	N/A	Dehghani et al. (2017)
Total atmospheric fallout (dry & wet deposition) Paris, France	118 particles/m^2^ d	Fibers >90% Fragments <10%	100–5,000 μm	Stereo microscope μFT-IR	N/A	Dris et al. (2015)
Atmospheric fallout Paris, France	2.1–355.4 particles/m^2^d	Fibers	50–4,850 μm	Stereo microscope μFT-IR	29% synthetic	Dris et al. (2016)
Indoor and outdoor air Paris, France	1,586–11,130 particles/m^2^d	Fibers	50–450 μm	Stereo microscope μFT-IR	PP	Dris et al. (2017)
Atmospheric deposition Hamburg, Germany	136–512 particles/m^2^d	Fragments >90% Fibers <10%	63–5,000 μm	μ-Raman	EVAC PAV PE PTFE	Klein and Fischer (2019)
Indoor and outdoor dust Major cities in China	212–120,000 mg/kg	Fibers Granule	N/A	μ-FT-IR	Nylon PC PE PEI PET PMMA PP PU	Liu et al. (2019a)
Suspended atmospheric fallout Shanghai, China	4.18 particles/m^3^	Fibers ~67% Fragment ~30% Granules ~3%	23–5,000 μm	Stereo microscope μ-FT-IR analysis	PAA PAN PE PES PET Rayon	Liu et al. (2019b)
Suspended atmospheric aerosols West Pacific Ocean	0–1.37 particles/m^3^	Fibers ~60% Fragments ~31% Granules ~8%	20 μm–2 mm	Stereo microscope μ-FTIR	PET PP PS PVA PVC	Liu et al. (2019c)
Alpine Snow (wet deposition) Austria	4,600–23,600 ng/l	N/A	N/A	Thermal desorption-proton transfer reaction-mass spectrometry (TD-PTR-MS)	PC PET PVC	Materić et al. (2020)
Atmospheric wet and dry deposition Nottingham, UK	0–31 particles/m^2^d	Fibers	38 μm–5 mm	FTIR	Acrylic PP PA Polyester	Stanton et al. (2019)
Atmospheric aerosols North Atlantic Ocean	0.86–1.04 g/cm^3^	N/A	10 nm-1 μm	μ-Raman spectroscopy	PC PE PP PS	Trainic et al. (2020)
Fresh falling snow Montreal, Canada	21,900 ng/l	Fragments	124–376 nm	Nanostructured laser desorption/ionization time-of-flight mass spectrometry (NALDI-TOF-MS)	PEG PE	Wang et al. (2021b)
Atmospheric deposition Yantai, China	115–602 particles/m^2^d	Fibers Films Foams	100–300 μm	Stereomicroscope μ-FT-IR analysis	N/A	Zhou et al. (2017)

Microplastics and nanoplastic have also been noted in the interactive dynamic and thermodynamic processes occurring between the atmosphere and the other environmental interfaces. For example, in a recent investigation, the supraglacial debris of the Forni Glacier (Italian Alps) reportedly contained 74.4 ± 28.3 MNPs/kg of dry weight sediments (Ambrosini et al., 2019). Other studies have also reported the presence of MNPs in fresh falling snow in Canada (21,900 ng/l) and Austria (23,600 ng/l; Materić et al., 2020; Wang et al., 2021b). However, higher MNP concentrations were found in the Arctic and European snow, ranging from 0 to 154,000 particles/l, indicating high MNPs transmission along with this atmospheric current (Bergmann et al., 2019). Similarly, very high concentrations of MNPs were observed in urban dust from Tehran metropolis, Iran (2933–20,167 particles/kg of dry dust), and indoor and outdoor dust from major cities in China (212–120,000 mg/kg; Dehghani et al., 2017; Liu et al., 2019a). Hence, considering the wide distribution of MNPs’ pollution across the different regions of the world, MNPs’ analysis should form an integral part of the routine air quality analysis in the future. Furthermore, MNPs in the atmosphere can ultimately find their way into different land organisms *via* respiration, most specifically humans. In contrast, epidemiological studies have connected MNPs air pollution to severe respiratory and cardiovascular repercussions (Zhang et al., 2020d).

**Figure 2:**
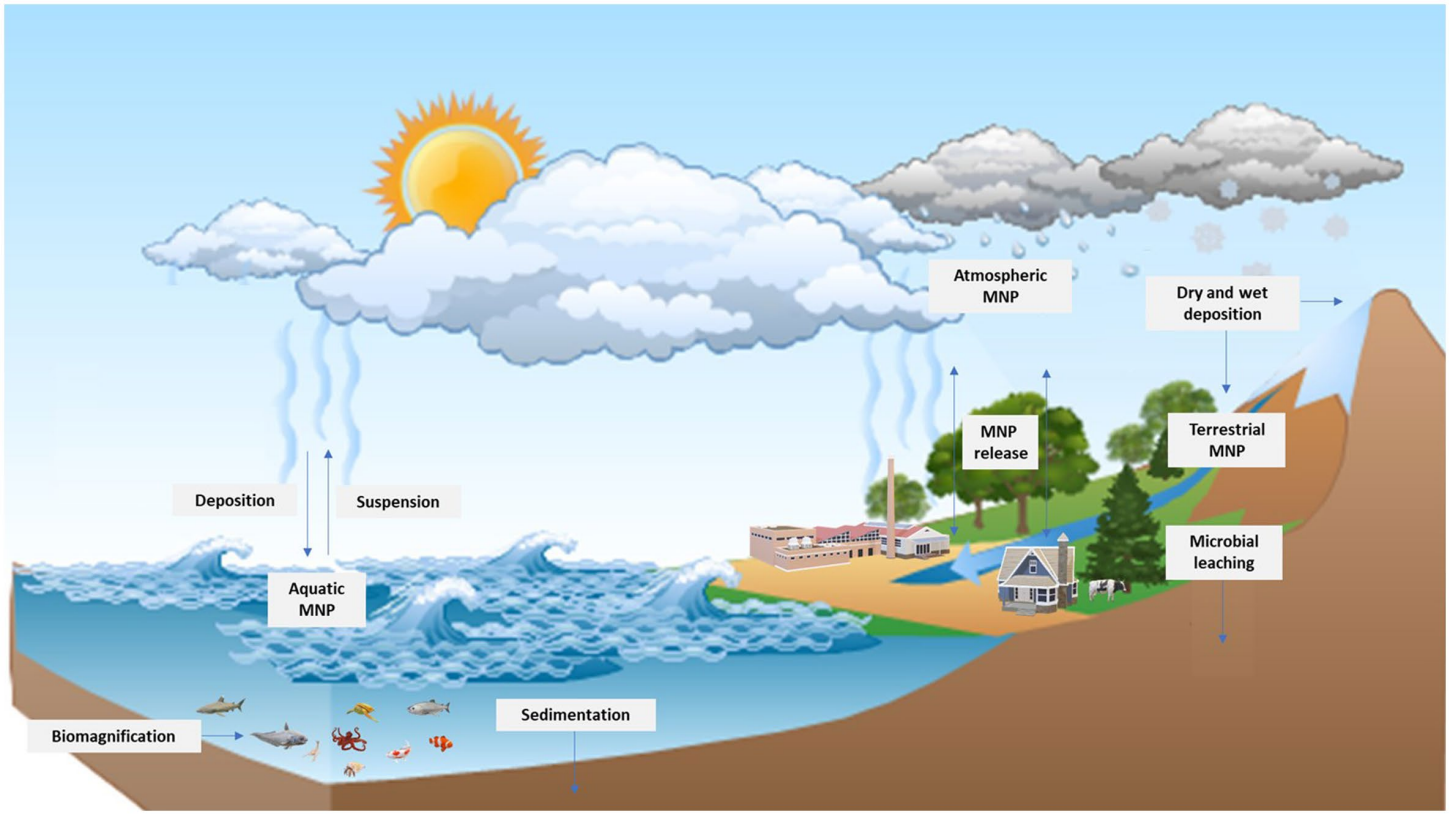
Conceptual model of the biogeochemical cycle of the MNPs.

As was previously noted, the atmospheric transportation of MNPs is a component of the dynamic cycle of MNPs in the environment, based on their exchange across the atmosphere, terrestrial, and aquatic ecosystems (Chen et al., 2020a). Furthermore, atmospheric transport is regarded to be an important pathway in the source–sink dynamics of plastic pollution in different ecosystems (Zhang et al., 2019. Although a significant deposition of plastic particles in the oceans has been ascribed to riverine and coastal discharge (Meijer et al., 2021), the pathway of MNPs transport from the terrestrial ecosystems to the marine environment and vice versa could be partly attributed to the atmospheric distribution (Liu et al., 2019c). The biogeochemical cycle of MNPs across the atmosphere, the aquatic, and the terrestrial ecosystems is depicted in Figure 2.

Like all atmospheric pollutants, it is believed that the mechanistic transport of MNPs is most likely *via* dispersion and deposition, as they probably become airborne and transported *via* wind deflation (González-Pleiter et al., 2021). In this regard, recent experimental and modeling studies have highlighted the atmospheric transportation of MNPs across various distances as well as across different ecosystems (Allen et al., 2019; Liu et al., 2019c; Wright et al., 2020). For instance, air mass trajectories revealed that MNPs were transported within a distance of 95 km, with the deposition observed in even relatively pristine areas (Allen et al., 2019). However, the transportation of MNPs beyond a distance of 100 km has also been recorded (Enyoh et al., 2019). Thus, the observations of MNPs in snows in remote locations such as the Arctic, Swiss Alps, and in metropolitan locations like Bremen, Germany, point to the spatial transportation of MNPs *via* the atmosphere (Bergmann et al., 2019).

The different factors that affect the atmospheric transportation of MNPs have been identified to include wind direction, particle dimension, rainfall, human activities, as well as population densities (Zhang et al., 2020d). Furthermore, the relationship between wind direction and the concentrations of airborne MNPs has been demonstrated in some studies (Abbasi et al., 2019, Browne et al., 2010, Chen et al., 2020a). For instance, in Hamburg (Germany), an increase in MNPs’ contamination was recorded with a direction change from westerly winds to southerly winds (Klein and Fischer, 2019), while higher abundances of airborne MPs were also recorded at downwind sites (Chen et al., 2020a). The characteristics of MNPs, particularly their sizes, shapes, and lengths are also major factors influencing the transport of MNPs *via* the atmosphere (Allen et al., 2019; Enyoh et al., 2019). In a recent study by Enyoh et al. (2019), it was demonstrated that smaller MNPs (<25 μm) dominated the sampled location, while larger-sized MNPs were relatively less recorded.

## Toxicological Effects of Mnps on Human Health

The processes leading to the formation of MNPs have been highlighted to alter the physicochemical properties of the minute particles such that their conductivity, particle size, reactivity, surface area, and strength differ significantly from their parent materials (Mattsson et al., 2015; Gonçalves and Bebianno, 2021b). Although there is still more to be learnt about the eventual fates of MNPs in various biological systems, especially in humans, it has, however, been noted that the biological reactivities of plastic particles like most other materials increase with decreasing particle size and surface area (Ferreira et al., 2019). As highlighted in the previous sections, MNPs have been demonstrated to affect different life forms across various ecosystems. However, it is important to highlight the potential effects of MNPs on humans, being at the top of many food chains. Furthermore, most plastics materials are often supplemented with additives to enhance their properties, such additives include plasticizers, coloring agents, as well as flame-retardation and UV-resistance chemicals (Kitahara and Nakata, 2020). These additives are also of small molecular size, similar to the MNPs. In addition, they are not chemically attached to the polymeric materials, increasing their likelihood of leaching into the surrounding environment and being transported through different food chains (Luo et al., 2020).

It has been demonstrated severally that smaller nanoplastics have a higher probability of gaining entrance and accumulating in different cells and tissues and subsequently affect the physiological activities of the cells and tissues. In this regard, different *in vivo* and *ex vivo* studies have demonstrated these aforementioned phenomena and also went further to explain the factors that might amplify or diminish the toxic effects of MNPs on living cells (Campanale et al., 2020). The effects of the size of MNPs on their entry into living systems have been demonstrated previously by Forte et al. (2016). The studies showed that the rate of cellular assimilation of polystyrene nanoplastics was inversely proportional to the size of the particles. In this regard, the MNPs sized 44 nm had higher uptake and toxic effects on the cell lines compared to 100-nm polystyrene MNPs (Forte et al., 2016). Furthermore, the unmodified plastic polymers were shown *in vitro* to specifically affect cell viability, inflammatory gene expression, and cell morphology of gastric cell lines (Forte et al., 2016). It has also been shown that the introduction of positive or negative charges to MNPs enhances their assimilation and toxicity to different cells. For instance, cationic polystyrene nanoplastics (60 nm) were shown to be uptaken by different cell lines including macrophage (RAW 264.7) and epithelial (BEAS-2B) cells inducing significant damages in the process highlighted. Results from a study by Bhattacharjee et al. (2014) also corroborated those shown previously by Xia et al. (2008) as cationic nanoparticles demonstrated higher toxicity than anionic ones in both studies. It could also be inferred from these studies that the toxic effects of MNPs on the cellular environment might result from induced oxidative stress, which leads to a cascade of undesirable cellular activities and eventual damage (Bhattacharjee et al., 2014; Guo et al., 2020). Hence, it is believed that MNPs can be accumulated in cells and tissues causing metabolic disorders and local inflammation (Hu and Palić, 2020). In this regard, the uptake, as well as cytotoxic effects of MNPs, has been demonstrated on various human cells lines including lung cells (An et al., 2020), intestinal cells (Stock et al., 2019), as well as cerebral and epithelial cells (Schirinzi et al., 2017).

**Figure 3:**
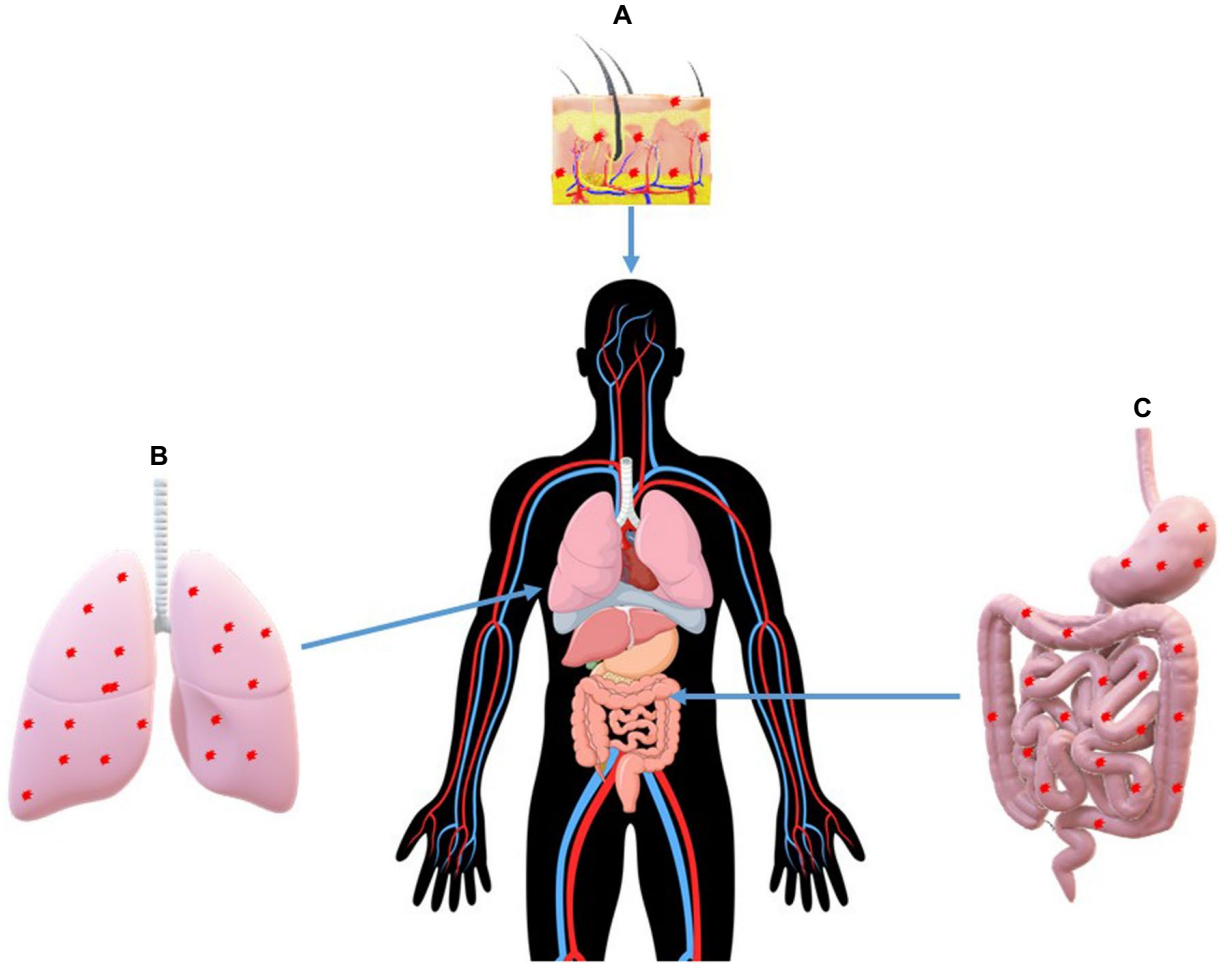
Primary paths of human exposure to MNPs: *via* (A) skin, (B) lungs, (C) digestive system.

The appearance of MNPs in humans and other higher organisms through different food chains has been a subject of intense scientific concern. Although there are yet to be conclusive data to establish the assimilation and metabolism of MNPs in the human body, different entry routes and mechanism of assimilation have been proposed as depicted in Figure 3. Alternative routes by which MNPs can be absorbed by humans, besides the food chain, have also been identified to include animal feeds (Karbalaei et al., 2020) and consumption of sea salt (Gündoğdu, 2018). Lately, a lot of scientific and public attention has been drawn toward the probability of the direct ingestion of MNPs, especially polyethylene terephthalate, polystyrene, and polypropylene MNPs (Lam et al., 2020; Senathirajah et al., 2021). For example, a most recent study has shown the presence of MNPs in approximately 80% of all the water bottle brands investigated (Makhdoumi et al., 2021). It was previously estimated that individuals who consume bottled water might be ingesting an additional 90,000 microplastics annually, compared to 4,000 microplastics for individuals who rely totally on tap water (Cox et al., 2020). Additionally, the study has also shown that the direct ingestion of MNPs by humans might also be possible *via* the consumption of alcohol, sugar, as well as tap water, as different levels of MNPs were discovered in these samples (Cox et al., 2020). Similar studies have also shown the presence of MNPs in beer, honey, milk, and some other beverages (Diaz-Basantes et al., 2020; Edo et al., 2021). However, the authors posited that the introduction of MNPs into these beverages is *via* the environment; for instance, many of these products are made using municipal water (Diaz-Basantes et al., 2020; Edo et al., 2021). They also added that the MNPs might have been transported by the bees into the hive or introduced by humans during honey processing.

The absorption of MNPs in mammals has been highlighted in different studies by estimating their bioavailability (Jani et al., 1990; Hillery et al., 1994). The oral bioavailability of polystyrene MNPs in rat mammalian models was estimated at 7%, while approximately 4% bioavailability was recorded in the blood, bone marrow, liver, and spleen (Jani et al., 1990). Higher bioavailability of the polystyrene MNPs (~10%) has also been recorded in the study by Hillery et al. (1994). The different bioavailability observed with the same MNPs in the two different studies has been ascribed to factors such as ageing time and surface modification (Shen et al., 2019). The effects of these factors have since been affirmed in subsequent studies (Kulkarni and Feng, 2013; Walczak et al., 2015). Recently, polystyrene MNPs were shown to cause cardiac fibrosis in rats by activating the Wnt/β-catenin signaling pathway and triggering cardiomyocyte apoptosis (Li et al., 2020c). Similarly, polystyrene MNPs were also demonstrated to induce blood–testis barrier disruption regulated by the MAPK–Nrf2 signaling pathway in rat models (Li et al., 2021). Even though humans have been exposed to MNPs from personal care products, especially skin cosmetics, there are currently no studies to demonstrate the dermal bioavailability of these particles. In this regard, Hwang et al. (2020) posited that human intake of MNPs from microbeads in personal care products could occur through skin absorption; however, their study could not substantiate this claim with any scientific experimentation, neither in animal models nor in cell lines.

It is also believed that the large surface areas and complex surface structures of MNPs, like other nanoparticles in general, would enhance their interaction with various cellular compounds including ions, lipids, proteins, and water (Chain, 2016). Hence, different MNPs have been shown to interfere with lipids metabolism and transportation in various organisms (Lu et al., 2018; Guo et al., 2020). Recently, MNPs have also been demonstrated to distort the structural integrity of proteins by changing the secondary structures (Hollóczki and Gehrke, 2019) and inducing protein misfolding (Hollóczki, 2021). Furthermore, proteins–nanoparticles interactions have also been shown to generate coronal protein rings (Gopinath et al., 2019), which significantly affect the endocytosis of nanoparticles in cells (Yee et al., 2021). The interaction of MNPs and metallic ions like iron ions has also been suggested to promote increased ion uptake, thus affecting the integrity and function of the membrane (Mahler et al., 2012). MNPs may also directly or indirectly impact human health by acting as vectors of environmental contaminants (Hartmann et al., 2017). The general nonpolar surfaces of MNPs, like their parent plastic polymer materials, mean that they can absorb and consequently transport other hydrophobic compounds, particularly persistent organic pollutants (POPs; Mattsson et al., 2018). However, it was shown that MNPs mostly act more as passive samplers instead of vectors for POPs even though the chemical transfer may still occur (Herzke et al., 2016).

## Biodegradation of Mnps

MNPs are difficult to degrade since they are discretely present in the environment, necessitating the use of effective waste management technology, which can be a powerful tool in combating the adverse implications, while, in the past few years, researchers have been focused on the degradation of MNPs using microorganisms, which is one of the potential alternative ways for plastics degradation (Pathak, 2017). Generally, the four major factors that interplay to culminate into microplastic degradation have been identified as biodegradation, hydrolysis, photodegradation, and thermooxidative degradation (Ghosh et al., 2013). Due to the presence of water in the aqueous habitats, it is expected that hydrolysis will play a more dominant role in influencing plastic biodegradation. Conversely, in terrestrial habitats, the ability of organisms to degrade plastic polymers is expected to be more influenced by heat (thermooxidative) and light (photodegradation). However, it has been observed that the interplay of the many environmental factors introduces many complexities into the degradation of plastic polymers in the different habitats. For example, the rate of hydrolysis of most plastic polymers has been described as insignificant in the ocean. Furthermore, the photodegradative effect is also significantly diminished in seawater due to the lower temperature and oxygen availability (Webb et al., 2013).

Biodegradation is the breakdown of complex polymeric materials by (micro) organisms, including archaea, bacteria, and fungi, into non-toxic products, which can be reintroduced into the biogeochemical cycles. Currently, not much is known about the specific mechanisms of microbial degradation of these MNPs. However, several researchers have proposed general mechanisms for this phenomenon, which involves biodeterioration, biofragmentation, assimilation, and mineralization, which are all executed *via* various bond cleavages and enzymatic activities (Ghosh et al., 2013; Amobonye et al., 2020).

Primarily the biodeterioration of MNPs, like other types of plastic, is initiated by the adherence of the microorganisms to the surface of the polymer to facilitate surface colonization. During this attachment, the chemical and physical actions of the microorganism, enhanced by the action of other biological agents and external environmental conditions (chemicals, light and temperature), result in the modification in the properties of the MNPs (Habib et al., 2020). Subsequently, the organisms depolymerize the deteriorated plastics *via* the actions of their extracellular enzymes and generate free radicals. Usually, the enzymatic degradation has been shown to produce the constituent oligomers or monomers, such as ethylene glycol and terephthalic acid, which can be further assimilated into the cell (Pathak, 2017). The assimilation of simpler intermediates into microbes has been described to be facilitated by many mechanisms in various organisms, including active and passive transports (Amobonye et al., 2020). Many biomolecules, including the ATP binding cassette family of proteins, monooxygenases, and porins, have been implicated in the assimilative transport of the plastic degradative intermediates (Hosaka et al., 2013; Gravouil et al., 2017). The final step involves the action of intracellular enzymes, which completely break down the assimilated into oxidized metabolites which includes CH_4_, CO_2_, H_2_O, and N_2_. However, the final products formed have been noted to be a function of the respiration condition of the microbe. In this regard, oxygen serves as the electron acceptor under aerobic conditions, resulting in the formation of CO_2_ and H_2_O (Priyanka and Archana, 2011). Under anaerobic conditions, however, other compounds such as carbon dioxide, iron, manganese, nitrates, and sulfates may serve as the terminal electron acceptors (Ahmed et al., 2018).

Microbes, including bacteria and fungi, from different environments have since been shown with the ability to degrade different MNPs to the point of mineralization (Sánchez, 2020; Li et al., 2020b). The potential of bacteria, especially from marine habitats, to colonize and degrade various MNPs has been highlighted by various studies. Recently, *Bacillus* and *Pseudomonas* species were predominant in the bacterial community colonizing MNPs in an estuary (Wu et al., 2020). This is in line with previous findings highlighting these two genera with the highest potential for plastic biodegradation (Wilkes and Aristilde, 2017; Amobonye et al., 2020). Similarly, various bacterial community assemblages were also described to colonize microplastics in a river, hence revealing the occurrence of potential plastic-degrading microbes (Niu et al., 2021). Recently, the potentials of fungal organisms in the degradation of petroleum-based MNPs have also raised some interest (Sánchez, 2020). For example, *Zalerion maritimum*, a marine fungus (Paço et al., 2017), and *Aspergillus flavus* isolated from an insect’s gut (Zhang et al., 2020c) are among the many fungi that have been studied for their MNPs’ degradative abilities. However, the environmental significance of the biodegradative action of microbes on MNPs and plastic, in general, is still a matter of conjecture. Various authors have argued that the microbial degradation rate is very low to the point of having no significant effects on the environmental rejuvenation efforts (Müller et al., 2012). This has increased the calls for research applying short-term experimental results to predict long-term degradation pathways and using computational methods to simulate MNPs degradation (Chamas et al., 2020).

## Mitigating the Effects of Mnps

Efforts from different angles are being devoted to attenuating the detrimental effects of plastic fragments arising from the current large-scale increase in plastic production and use. Evaluating the impacts of MNPs in the environment on an economical scale is inherently difficult because of the wide knowledge gaps. However, plastic wastes, including macro-, meso-, micro- and nanoplastics, have been estimated to cause a global annual financial loss of ~13.3 billion US dollars (da Costa, 2018). These financial implications have been identified to include costs resulting from the direct consequence of these plastic litters on life forms as well as clean-up and prevention costs (Lee, 2015). Hence, the onus has been on scientists, the industries, government authorities, and the public worldwide to reverse the continuous accumulation of MNPs in the different environments. Although there have been little or no data to show the damaging effects of MNPs on higher animals, especially humans, it is widely believed that these particles will adversely affect all forms of life in the long run. Hence, in this regard, biological experiments should not be restricted to single/lower organisms or at individual levels but should be extended to account for the higher members of the different food chains. This is significant because the clearance and resistance ability of lower organisms to MNPs and other xenobiotics are supposed to vary remarkably compared to higher organisms as much as their metabolic systems vary. For example, it has been posited that organisms at both lower and higher trophic levels should be exposed to the same MNPs containing environments to better evaluate the cumulative effects on behavioral changes, development, reproduction, survival, and toxicity (Shen et al., 2019). Furthermore, there is the need to further explore MNPs’ toxicity by extending laboratory studies to real-time environment conditions through improved pollutant risk assessment methodologies to predict MNPs’ exposure and their associated pathophysiological risk. These would facilitate the determination of effective concentrations as well as increase the accuracy of predicting the real-time environmental risks associated with MNPs (Anbumani and Kakkar, 2018). Furthermore, system-based analysis of the MNPs’ toxicological effects under field and laboratory exposures will give more insights into the necessary steps for remediation and pave the way for a more sustainable ecosystem.

Biotechnology is currently being explored through different novel approaches to manage and minimize the risks posed by MNPs’ accumulation in the environment. One of the most feasible approaches is making bioplastics, which are more sustainable and environmentally friendlier than fossil-fuel-based plastics. These bioplastics, which include polylactic acid, poly-3-hydroxybutyrate, polyhydroxyalkanoates, polyhydroxyvalerate, polyhydroxyhexanoate, and polyamide 11, are principally derived from renewable organic materials and hence can be significantly degraded by different microbes and their enzymes. Furthermore, the remarkable hydrophilic nature of these bio-based and biodegradable polymers has been identified as the key factor that enables their biodegradation and hydro-degradation (Filiciotto and Rothenberg, 2021). Furthermore, it has been demonstrated in various studies that microorganisms can effectively utilize these bioplastic polymers as nutrients sources, metabolizing them and converting them into simpler compounds as against the MNPs from other plastics. Hence, these “new” plastics are believed to have the potential of totally replacing the fossil fuel-based plastics in the coming future as they possess similar properties and functionalities with their older counterparts and are expected to contribute less burden to the different ecosystems and the environment in general (Ogunola et al., 2018). In addition, various efforts are also being dedicated to the degradation of recalcitrant synthetic plastic polymers, which currently accounts for most of the plastic wastes burdening the environment. In this regard, the biodeterioration, biofragmentation, assimilation, and mineralization of these petrochemical-based polymers and their miniaturized components by various actinomycetes, bacteria, fungi, and insects as well as different microbial enzymes are being vigorously investigated (Amobonye et al., 2020), and these have been well discussed in the previous section of this paper.

Besides these aforenoted scientific efforts, other mitigating measures revolve around reducing the generation of plastics wastes and preventing them from entering the different ecosystems as much as possible. One of these measures involves strategies by various governments to gradually/outrightly ban the general sale/use of plastic bags and the industrial application of microplastics in cosmetic products. The ban or/and restriction on single-use plastic bags has been noted to be effective in the reduction of plastic wastes accumulation in the more than 30 countries worldwide that have partially or completely effected these bans (Ogunola et al., 2018). For example, the imposition of the plastic bags tax in Portugal was estimated to result in a remarkable 400% reduction in the number of plastic bags consumed per person per shopping trip (Martinho et al., 2017). However, the early enforcers of this ban have been mainly from European countries, especially Denmark and Germany, which have had this ban for close to 30 years (Xanthos and Walker, 2017). However, a few developing countries, including India and some Latin American countries, have also identified the need for the control of plastic wastes and have introduced different bans/restrictions across various government levels (Vimal et al., 2020). For instance, in 2017, the Indian capital city of Delhi banned all forms of disposable plastics, while in 2016, the southern state of Karnataka also imposed a total ban on single-use plastic items (Radha, 2019).

Ecolabeling has also been identified as a proactive and invaluable tool to effectively prevent or reduce plastic pollution (Anagnosti et al., 2021). The principal objectives of this approach are to minimize the adverse environmental impacts of products and increase consumers’ environmental consciousness, consequently increasing the chances of them making more environmentally sustainable choices. Scientific studies focused on assessing public perceptions about plastic pollution have shown that a large proportion of the public was ignorant of the MNPs and their effects on the environment, as against other issues such as climate change and ocean acidification (Ogunola et al., 2018). Hence, it is believed that public awareness programs and educational outreaches by both governmental and non-governmental (NGOs) organizations will remarkably promote behavioral and perfectional changes that will eventually lead to reduced indiscriminate use and disposal of plastic wastes.

On the industrial side, it is believed that reducing the use of microplastic beads in cosmetic products and other industrial products will help mitigate these efforts against MNPs. In this regard, mandatory phase-out of microplastic beads through legislative ban by governments and their regulatory agencies will enhance the universality and effectiveness of this approach (Anagnosti et al., 2021). Recycling is also a sustainable approach to reduce the potential damages arising from MNPs; this is because the huge amount of discarded plastic wastes in landfills and natural habitats worldwide at one point end up as MNPs. However, the recycling rate for plastic waste varies in different countries, being very low in developing countries and significantly higher in developed countries. This is mainly because the production of recycled plastic is not economically competitive and thus attracts minimal investments. However, actions such as imposing taxes on the use of virgin plastics, awareness of the environmental benefits of recycled plastics, and government incentives toward their production will aid in driving the market for recycled plastics (Calero et al., 2021).

## Conclusion

Microplastics and nanoplastic, miniaturized products of plastic materials, have been observed to move great distances around the planet due to their distinct properties, including buoyancy, durability, lightweightness, and shapes. However, terrestrial ecosystems have been identified as the major sources and transport pathways of MNPs into the aquatic environments as well as the atmosphere. The heightened accumulation of MNPs in these environments strengthened by their distinct characteristics has ensured that they gain access into microbes and other organisms at the lower end of the food chain, especially marine biota such as phytoplankton and eventually find their ways up the food chains. On entering these organisms, they accumulate in their various organelles and organs, eliciting different toxic effects mainly *via* oxidative stress. However, the different scientific efforts aimed at mitigating the damaging effects of MNPs on life forms have not been as effective as expected due to their rate of increase in the environments and the complexities of the different organisms in the food chains. The associated timescale required to demonstrate the biodegradation of MNPs has also been identified as a major obstacle. Hence, given the dearth of understanding regarding the ecotoxicological effects and environmental fate of MNPs, either from primary or secondary sources, it is necessary to implement appropriate scientific methods with higher efficiency to assess their potential environmental risks and toxicological effects across different trophic layers. Researchers are also obliged to efficiently quantify the degradation rates from large plastic particles to MNPs and the mechanisms behind these degradation processes. Furthermore, there is also the urgent need to develop and implement sound legislation and regulations regarding MNPs and plastics materials in general. Efforts at standardizing monitoring methods, identifying technical and waste management gaps, and encouraging recycling will all go a long way in mitigating the detrimental effects of MNPs. This review is expected to help understand the enormity of MNPs’ problems and give valuable insights into the management of MNPs in both developing and developed countries of the world.

## Author Contributions

AA and PB contributed to conceptualization, investigation, formal analysis, and writing – original draft. SR done writing – review and editing. SS performed resources and supervision. SP was involved in resources, writing – review and editing, supervision, and funding acquisition. All authors contributed to the article and approved the submitted version.

## Conflict of Interest

The authors declare that they have no known competing financial interests or personal relationships that could have influenced the work reported in this paper.

## Publisher’s Note

All claims expressed in this article are solely those of the authors and do not necessarily represent those of their affiliated organizations, or those of the publisher, the editors and the reviewers. Any product that may be evaluated in this article, or claim that may be made by its manufacturer, is not guaranteed or endorsed by the publisher.

## Footnotes

^1^ http://apps.isiknowledge.com

^2^ http://www.ncbi.nlm.nih.gov/pubmed

^3^ http://www.sciencedirect.com/

^4^ http://www.scopus.com/
